# A Comprehensive Analysis of Body Mass Index Effect on *in Vitro* Fertilization Outcomes

**DOI:** 10.3390/nu8030109

**Published:** 2016-02-23

**Authors:** Veronica Sarais, Luca Pagliardini, Giorgia Rebonato, Enrico Papaleo, Massimo Candiani, Paola Viganò

**Affiliations:** 1 Obstetrics and Gynecology Unit, San Raffaele Scientific Institute, 20132 Milano, Italy; sarais.veronica@hsr.it (V.S.); g.rebonato@studenti.unisr.it (G.R.); papleo.enrico@hsr.it (E.P.); candiani.massimo@hsr.it (M.C.); 2 Division of Genetics and Cell Biology, San Raffaele Scientific Institute, 20132 Milano, Italy; vigano.paola@hsr.it

**Keywords:** BMI, assisted reproduction, infertility, oocyte

## Abstract

The effect of a raised body mass index (BMI) on the outcome of assisted reproduction technology (ART) still represents a controversial issue. Even less clear is whether BMI acts with a potential detrimental effect on IVF outcomes via a deleterious effect on innate quality of oocytes or on the environmental milieu within the uterus. With the aim to better understand the mechanisms underlying the potential deleterious effect of an increased BMI on IVF outcomes, we have evaluated the effects of female BMI on number and quality of retrieved oocytes, fertilization rate, embryo score and incidences of ongoing pregnancy and live births among couples undergoing IVF in an Italian population. Data from 1602 women who underwent their first IVF cycle were retrospectively analyzed. A significantly reduced percentage of mature oocytes when comparing obese (BMI ≥ 30 kg/m^2^) and normal-weight patients (BMI = 18.50–24.99 kg/m^2^) was found. After adjusting for maternal age and other confounders, odds for ongoing pregnancy rate showed no differences across different BMI categories. However, a significant increased odds ratio (OR) could be observed for miscarriage rate in patients with BMI ≥ 25 (OR = 2.5; *p* = 0.04). These results should be taken into account in order to define optimal strategies for overweight and obese patients referring to ART procedures.

## 1. Introduction

In the United States, almost two thirds of women and three fourth of men are overweight or obese, as are nearly 50% of women of reproductive age [1,2,3,4]. Among European countries, more than four Italian subjects out of 10 (42%) were overweight or obese in 2014 [5]*.* Obesity is well known to increase the rate of miscarriage regardless of the mode of conception [6] and is strongly associated with pregnancy and perinatal complications, including gestational diabetes, preeclampsia, preterm delivery, stillbirth, cesarean section, shoulder dystocia, fetal distress, and small, as well as large, for gestational age of infants [7,8]. Moreover, there is an increased prevalence of infertility among overweight and obese women [9,10]. Infertility affects one in seven couples and a significant proportion of these cases are thought to be either directly or indirectly related to obesity. Obese women in the general population have a lower chance of conception within one year of stopping contraception compared with normal-weight women. The combination of infertility and obesity confers some real challenges about the short and long term management of these women [11].

The mechanism through which obesity is thought to affect female reproductive function is complex. Adiposity increases peripheral aromatization of androgens to estrogens with a concurrent decrease in the hepatic synthesis of sex-hormone-binding-protein (SHBG). This is associated with a hypersecretion of luteinizing hormone (LH) and an increase of androgen to oestrogen ratio with a consequent overall altered endocrine environment leading to impaired folliculogenesis. The overall adiposity is further associated with changes related to inflammation, coagulation and fibrinolysis [12], as well as with insulin resistance and metabolic syndrome [13].

However, there is conflicting evidence with regard to the effects of a raised body mass index (BMI) on the outcome of assisted reproduction technology (ART). In obese women it is well known that increased doses of medications to induce ovulation or to stimulate the ovaries for *in vitro* fertilization (IVF) are needed. Several large, retrospective analyses confirm that obesity impairs ovarian responsiveness to gonadotropin stimulation [14,15,16,17] while there are controversial data on the effect of obesity on live birth rate following ART [18]. A systematic review of 27 IVF studies, 23 of which were retrospective, showed that overweight women (BMI > 25 kg/m^2^) undergoing IVF have a 10% lower live birth rate than normal weight women (BMI < 25 kg/m^2^) [19]. Other studies have reported no adverse effects of an increased BMI on IVF pregnancy outcomes [17,20,21,22,23]*.* Even less clear is whether BMI acts with a potential detrimental effect on IVF outcomes via a deleterious effect on innate quality of oocytes or on the environmental milieu within the uterus [24].

There are some considerations to be done in this context. First, most of the studies addressing this issue refer to US population that is known to be different from the European populations. Second, no study has specifically evaluated IVF outcomes among Italian obese women that represent a unique population in terms of nutrition style and dietary pattern. Thus, with the aim to better understand the mechanisms underlying the potential deleterious effect of an increased BMI on IVF outcomes, the objective of our study was to assess the effects of female BMI on number and quality of retrieved oocytes, fertilization rate, embryo score, and incidences of ongoing pregnancy and live births among couples undergoing IVF in an Italian population.

## 2. Experimental Section

### 2.1. Patients and Methods

In the present analysis, we have considered 1602 IVF or intracytoplasmic sperm injection (ICSI) cycles. All patients were evaluated and treated at San Raffaele Hospital, a large university-affiliated infertility center. Our study represents a retrospective chart review of consecutive ART cycles performed at the Center from January 2012 to December 2014, using the electronic medical record database. Women included were aged 20–45 years, were having their first IVF or ICSI cycle using autologous oocytes and had a BMI recorded in their electronic medical chart. Height and weight were measured with standardized protocols. A wall mounted stadiometer measured height to the nearest 0.5 cm^2^ and weight was measured using calibrated scales to the nearest 0.1 kg. Body mass index was then calculated and was defined as weight in kilograms divided by the square of their height in meters (kg/m^2^). The most recent World Health Organization (WHO) classification of BMI categories was used to divide our population into four groups: <18.50 kg/m^2^ (underweight); 18.50–24.99 kg/m^2^ (normal weight); 25.00–29.99 kg/m^2^ (overweight); ≥30 kg/m^2^ (obese; due to the low number of cases, we joined class I with class II/III obesity categories) [25]. Patients were selected for IVF or ICSI cycle according to standard accepted indications [26]. Patients underwent ovarian stimulation with a standard agonist or antagonist protocol. These therapeutic regimens included: pituitary down-regulation with long luteal leuprolide acetate (Decapeptil^®^, Ipsen Pharma, Paris, France; Fertipeptil^®^, Ferring, Saint Prex, Switzerland); antagonist short protocol involving the use of GnRH antagonist (Cetrotide^®^, Serono, Geneva, Switzerland; Orgalutran^®^, MSD, Whitehouse Station, NJ, USA) when a lead dominant follicle measured ≥14 mm; a short flare up protocol introducing the GnRH agonist on day 2 of the cycle. The gonadotropins used included human menopausal gonadotrophins or purified urinary FSH, recombinant LH or recombinant FSH. The FSH starting dose and the type of protocol were determined according to ovarian reserve assessment, patient’s age, *i.e.*, basal hormonal status and antral follicular count, indication to IVF. The dosage of gonadotrophins varied during stimulation according to the patient’s ovarian response and ranged from 150 to 450 IU administered daily. Cycle monitoring was conducted with ultrasonography ovarian transvaginal and serum estradiol measurements and the frequency of patient’s monitoring was dependent of the ovarian response to ovarian hyperstimulation [27,28]. Human chorionic gonadotrophin (hCG) for ovulation trigger was administered to those patients who had at least one mature follicle ≥17 mm. Vaginal oocyte retrieval was performed 36 h after the administration of hCG or recombinant hCG by transvaginal ultrasonography-guided needle aspiration under anesthesia. IVF and/or ICSI cycle was performed by standard techniques [29,30,31]. Embryos were transferred to the uterus either 3 days (cleavage stage) or 5 days (blastocyst stage) after oocyte retrieval. The number of embryos transferred was established according to the American Society for Reproductive Medicine guidelines [32].

We decided to measure the live birth rate as primary outcome of our study which was defined by the delivery of a live infant at >23 weeks of gestation (as reported in our databases). Secondary outcomes were ongoing pregnancy, spontaneous abortion, number of oocytes retrieved and percentage of mature oocytes retrieved in various BMI categories. Ongoing pregnancy was confirmed by the ultrasonographic presence of an intrauterine sac with fetal heartbeat at 12 weeks of gestation. Spontaneous abortion was defined as loss of clinical pregnancy before 20 weeks of gestation. For each patient, the following information were obtained: age of both partners, baseline serum follicle-stimulating hormone (FSH)/thyroid-stimulating hormone (TSH)/anti-müllerian hormone (AMH) levels, current number of daily cigarettes smoked, antral follicle count, serum 25-hydroxy-vitamin D (25(OH)D) levels, menstrual cycle length, duration of infertility, parity status, cause of infertility, duration of stimulation, total dose of gonadotrophins used, peak serum estradiol (E_2_) level, number of oocytes retrieved, number of mature oocytes, use of IVF or ICSI, fertilization rate, number of embryos transferred, day of transfer, quality score of transferred embryos and blastulation rate.

To calculate the embryo quality score we first collected retrospective data of all patients in our center who transferred a single embryo and that have collected information about the presence of clinical pregnancy. Day 3 and day 5 embryos were evaluated according to the Istanbul consensus workshop on embryo assessment [33] and a regression model evaluating the embryo implantation potential was created. Based on the coefficients of the regression model, a score system from 1 to 3 was defined. This score system was then applied to all transferred embryos of the present study; the quality score of embryos was defined as the mean value of all the transferred embryos. Blastulation rate was calculated excluding patients with embryo transfer or embryo freezing in day 3, using as numerator the total number of blastocysts obtained and as denominator the number of fertilized oocytes. Ongoing pregnancy rate was calculated on the total number of patients undergoing embryo transfer procedures (*n* = 1037), after excluding patients who cryopreserved the totality of the embryos, that failed to retrieve oocytes or that have no eligible embryos for transfer. Data for gestational age and birth weight were obtained for 266 patients. Data collection followed the principles outlined in the Declaration of Helsinki; all women undergoing IVF/ICSI cycles routinely provide informed consent for their clinical data and anonymized records to be used for research purposes in general. Local Institutional Review Board approvals for the use of clinical data for research studies were obtained.

### 2.2. Statistical Analysis

Continuous variables are presented as mean ± SD, whereas categories variables are presented as frequency and percentages. Baseline characteristics of the four BMI groups were compared with the use of Pearson chi-square test for variables categories and one-way analysis of variance for continuous variables, using Tukey’s *post hoc* test for multiple comparisons. Outcomes of cycles were compared between BMI groups with the use of logistic regression to calculate the ORs, with their 95% confidential interval (95% CI). We performed adjustment for the following potential confounders: maternal age, paternal age, smoking, baseline FSH, number of oocytes retrieved, number of embryo transferred, day of embryo transfer, embryo quality score and cause of infertility. Statistical tests were performed using SPSS v.19 (IBM Corp., Armonk, NY, USA). All tests were two sided, with a significance level set at 0.05. Statistical power analysis was performed using G*power 3 [34] and revealed that the study has a power equal to 80% of detecting differences in live birth rate larger than 10% (assuming a type I error of 0.05).

## 3. Results

A total of 1602 patients who underwent their first fresh ART autologous cycle at our Infertility Center were included in the study. These patients were divided into four BMI categories: <18.50 kg/m^2^ (*n* = 142; 8.8%); 18.50–24.99 kg/m^2^ (*n* = 1164; 72.7%); 25.00–29.99 kg/m^2^ (*n* = 225; 14.0%) and ≥30 kg/m^2^ (*n* = 72; 4.5%). Baseline women characteristics are presented in Table 1.

No statistical differences across BMI groups were found for female age, male age, smoking, AMH, AFC and parity. Women with BMI ≥ 30 kg/m^2^ showed significantly lower levels of day 3 FSH when compared with all the other BMI categories. Moreover, in line with previous observations [35], both overweight and obese women presented lower concentrations of 25(OH)D when compared to normal-weight women. A longer duration of infertility was observed for overweight patients when compared to underweight and normal-weight women, while obese patients showed statistically higher duration of infertility only when compared to underweight women. Finally, significant differences in causes of infertility were observed among BMI categories. Overweight women showed a significantly lower risk of idiopathic infertility while an increased risk of ovulatory disorders and a decreased risk of poor ovarian response were observed for underweight women when compared to normal-weight patients. Characteristics of cycles are summarized in Table 2.

No statistical differences were reported across BMI groups for duration of stimulation, total doses of gonadotropin required, ovarian stimulation protocol, E_2_ peak serum level, number of oocytes retrieved, percentage of cycles without oocytes, percentage of ICSI cycles, fertilization rate, embryo quality score, day of embryo transfer, number of embryos transferred and blastulation rate. A significantly reduced percentage of mature oocytes was reported when comparing obese and normal-weight patients, resulting in a significant lower number of oocytes used for IVF/ICSI procedures. Pregnancy outcomes are presented in Table 3.

After adjusting for maternal age and other confounders, odds for ongoing pregnancy rate showed no differences across different BMI categories. No statistically significant differences were observed neither for miscarriage nor for live birth rates, although a trend could be observed with increased ORs for miscarriage and lowered ORs for live birth in underweight, overweight and obese patients when compared to normal-weight women. However, a significant increased OR could be reported for miscarriage in overall patients with BMI ≥ 25 (OR = 2.5; 95% CI 1.02–6.14; *p* = 0.04). Finally, no significant difference was observed when comparing gestational age and birth weight in singleton births among the four BMI categories.

## 4. Discussion

Our study highlights the consequences of female obesity on IVF outcomes in an Italian IVF center. No statistical difference was found among BMI groups in total FSH doses, number of oocytes retrieved and quality of embryos obtained. In line with some previous observations [35,36], we showed that overweight and obese infertile women had basal serum level of FSH and vitamin D concentration significantly lower than women with normal BMI. In our study, we have found a similar need in total dose of FSH used for stimulation among groups while most, although not all, studies conducted in obese women undergoing IVF cycles reported the opposite observation [22,37,38,39,40,41,42]. It should be considered in this regard that most of the studies performed so far refer to US women but there is a large difference in mean BMI observed between Italian and US populations. Moreover, the frequency of polycystic ovarian syndrome (PCOS) is quite different between the two ethnicities. Although obesity has been reported as being present in only 40% ± 50% of women with PCOS [43], only 38% of Italian PCOS women had a BMI > 27 kg/m^2^, hence, it is possible that, in the USA, the prevalence of obesity among women with PCOS may be higher. Thus, it is likely that genetic and other lifestyle factors play a major role in supporting our observation [44].

Importantly, we have found a significantly lower number of MII oocytes in women with BMI ≥ 30 kg/m^2^ than women with normal BMI. This result was already found in experiments conducted in mice fed with a high fat obesogenic diet (HFD), reporting oocytes with delayed maturation and decreased developmental competence [45]. It has been demonstrated that these problems with oocyte function are a direct result of a mitochondrial dysfunction. In fact, an HFD leads to abnormalities of mitochondrial morphology, mitochondrial distribution within the oocyte, metabolism and spindle formation within the oocyte [46].

This lower number of MII oocytes, however, did not seem to impact either on the number and quality of embryo transferred or on the clinical pregnancy and live birth rate among different groups, thus again supporting the limited effect of BMI in the Italian population. These findings are however controversial in the literature. A recent retrospective study of 1721 first IVF cycles found a lower clinical pregnancy rate in class I-III obese patients and a lower live birth rate in class III obese patients only [47]. Another retrospective study of 6500 IVF cycles has also demonstrated decreased implantation, clinical pregnancy and live birth rates in obese patients, which persisted after controlling for age, cycle number and gonadotrophin dose [48]. Considering 8457 first IVF cycles, Lintsen *et al.* [49] have shown a significantly lower live birth rate per cycle in women with BMI > 27 Kg/m^2^. Conversely, several studies did not demonstrate statistically differences in live birth between normal and obese patients but the majority of these studies had much smaller sample sizes [21,50,51]. We indeed cannot exclude not to be able to observe a significant reduction in pregnancy rate for the limited number of obese patients in our series. Miscarriage rate, on the contrary, was found to be significantly increased in patients with BMI ≥ 25 Kg/m^2^. An increased risk of miscarriage is reported in various studies in overweight and obese women after spontaneous conception [52], ovulation induction [53], and IVF [54,55]. The reasons for an increased risk of miscarriage among overweight or obese women have been debated. It has been suggested that this could possibly be due to the higher prevalence of PCOS among overweight and obese women. Moreover, folliculogenesis and poor oocyte quality in obese women have been suggested as possible causes [56]. Evidence supporting this theory has recently been found by analyzing heterologous fertilization cycles, with the effect of obesity overcome when donor oocytes are used in obese women [57].

Our results are limited mainly by the retrospective nature of the study, which prevented us controlling for confounders such as lifestyle characteristics (physical exercise and dietary habit) or missing data and limited the possibility of causal inferences. However, the high number of patients and the presence of detailed clinical information allowed us to partially compensate these limits. This in fact represents one of the largest studies conducted in a European population, with an 80% power of detecting differences larger than 10% in live birth rate.

Another limitation is represented by the fact that this is a single center study. Our data have been obtained from a single institution and they lack the advantage of a multicentric study. More specifically, Luke *et al.* [58] analyzed data from 345 clinics, in cycles that resulted in embryo transfer and included more than just the first ART cycle. In contrast, the present findings were derived from a single institution where practice consistency can be assured and included all first-time cycling patients, which enabled us to evaluate cancellation rates and decrease the over-representation of patients with repeated failures.

Some clinical implications may derive from our study. Appropriate counselling to encourage weight loss through dieting and exercise may help these patients but this strategy might be very time consuming. As number and quality of embryo are not severely affected by an increased BMI while the maintenance of pregnancy seems to represent an issue in these women, a possibility to increase the chance of success could be the cryopreservation of embryos after the ovarian stimulation in anticipation of the patient’s weight loss (“freeze all” strategy). The improvements made in cryopreservation techniques have led to few or no detrimental effects to the embryo [59] and results from two randomized clinical trials [60,61] comparing the IVF outcomes of fresh ET and elective frozen embryo transfer (FET) showed better results for the latter. Additionally, the obstetric and perinatal outcomes appeared to be similar or even better [62] in FET compared to fresh ET. When comparing the risk of major congenital anomalies between children conceived after fresh ET and FET, no differences between the techniques were shown [63]. However, an increased risk of macrosomia in singletons born after FET when comparing to fresh embryo transfer was reported [64].

Although results from the present study would suggest that even this cause of reduced fertility might actually benefit of a “freeze all” strategy, evidence from randomized clinical studies of a “freeze-all” policy in obese women are needed in order to confirm the effectiveness of this strategy.

## Figures and Tables

**Table 1:** Participant characteristics by female BMI.

Variable	<18.50	18.50–24.99	25.0–29.99	≥30	*p* Value
	(*n* = 141)	(*n* = 1164)	(*n* = 225)	(*n* = 72)	
Female Age (years)	36.15 ± 3.95	36.86 ± 4.31	36.51 ± 5.01	37.16 ± 4.45	0.20
Male Age (years)	39.20 ± 5.22	39.51 ± 5.34	39.43 ± 5.67	39.67 ± 5.10	0.91
BMI (Kg/m^2^)	17.66 ± 0.65	21.28 ± 1.73	26.79 ± 1.29	32.53 ± 2.26	n/a
Current smoking (*n* cigarettes/day), *n* (%)					
0	115 (81.6)	937 (80.5)	187 (83.1)	63 (87.5)	
1–5	11 (7.8)	108 (9.3)	12 (5.3)	3 (4.2)	
≥6	15 (10.6)	119 (10.2)	26 (11.6)	6 (8.3)	0.38
Day 3 FSH (pg/mL)	8.32 ± 4.45	7.90 ± 3.74	7.29 ± 3.19	5.85 ± 2.44	<0.001 ^a,b,c,d^
TSH (µU/mL)	1.83 ± 1.02	2.01 ± 1.11	1.97 ± 1.70	2.10 ± 1.21	0.31
AMH (ng/mL)	2.54 ± 2.60	2.17 ± 2.12	2.46 ± 2.39	2.03 ± 2.17	0.10
AFC (*n*)	9.37 ± 5.50	9.43 ± 5.57	9.40 ± 6.04	10.24 ± 7.87	0.81
25(OH)D (ng/mL)	21.81 ± 9.09	23.84 ± 12.27	19.67 ± 10.00	18.04 ± 4.66	<0.001 ^c,e^
Menstrual cycle lenght (days)	28.30 ± 6.16	28.91 ± 6.34	29.37 ± 7.29	31.24 ± 10.15	0.02 ^b,c^
Duration of infertility (months)	38.76 ± 21.67	42.85 ± 27.11	55.39 ± 35.23	52.00 ± 37.23	<0.001 ^a,b,e^
Parity, *n* (%)	36 (25.5)	424 (36.4)	81 (36.0)	21 (29.2)	0.06
Cause of Infertility, *n* (%)					
Male factor	0.81 (0.51–1.27)	1.00	1.33 (0.94–1.88)	1.39 (0.79–2.44)	
Tubal factor	0.80 (0.38–1.69)	1.00	1.33 (0.79–2.26)	0.60 (0.18–1.94)	
Poor ovarian response	0.41 (0.19–0.90) ^f^	1.00	0.57 (0.32–1.01)	1.82 (0.96–3.43)	
Ovulatory disorders	1.70 (1.08–2.67) ^f^	1.00	1.47 (0.99–2.19)	1.27 (0.65–2.49)	
Endometriosis	0.95 (0.46–1.94)	1.00	1.39 (0.82–2.36)	-	
Idiopathic	1.20 (0.83–1.73)	1.00	0.62 (0.44–0.87) ^g^	0.73 (0.43–1.26)	
Mixed	1.23 (0.55–2.73)	1.00	2.22 (0.93–5.32)	1.15 (0.33–4.08)	

Notes: Data presented as mean ± SD, *n* (%) or OR (95% CI) (BMI category 18.5–24.99 was used as reference category). ^a^ Pairwise comparison revealed a statistically significant difference between the first and the third BMI categories; ^b^ Pairwise comparison revealed a statistically significant difference between the first and the fourth BMI categories; ^c^ Pairwise comparison revealed a statistically significant difference between the second and the fourth BMI categories; ^d^ Pairwise comparison revealed a statistically significant difference between the third and the fourth BMI categories; ^e^ Pairwise comparison revealed a statistically significant difference between the second and the third BMI categories; ^f^ *p* < 0.05; ^g^ *p* < 0.01. n/a not applicable.

**Table 2:** Cycle characteristics by female BMI.

Variable		>18.50	18.50–24.99	25.0–29.99	≥30	*p* Value
		(*n* = 141)	(*n* = 1164)	(*n* = 225)	(*n* = 72)	
Duration of stimulation (*d*)		10.35 ± 2.17	10.01 ± 2.55	10.09 ± 2.19	10.52 ± 1.72	0.53
Total dose of gonadotrophin (IU)		2439 ± 1403	2658 ± 1406	2573 ± 1029	2788 ± 1222	0.58
Ovarian stimulation protocol *n* (%)						
GnRH antagonist		101 (71.6%)	842 (72.3%)	180 (80.0%)	53 (73.6%)	
Long		32 (22.7%)	262 (22.5%)	37 (16.4%)	16 (22.2%)	
Others		8 (5.7%)	60 (5.2%)	8 (3.6%)	3 (4.2%)	0.41
E2 on hCG day (pg/mL)		1823.07 ± 969.29	1716 ± 1092	1883 ± 1354	1571 ± 872	0.17
Retrieved oocytes (*n*)		8.26 ± 5.87	8.17 ± 6.09	7.88 ± 5.83	6.22 ± 4.73	0.06
MII (%)		74.56 ± 21.93	76.93 ± 24.75	73.15 ± 24.96	67.24 ± 30.55	0.005 ^a^
Cycles without oocytes *n* (%)		12 (8.5%)	98 (8.4%)	20 (8.9%)	10 (13.9%)	0.46
Used oocytes (*n*)		5.61 ± 3.94	5.99 ± 4.12	5.62 ± 4.29	4.19 ± 3.43	0.003 ^a^
ICSI (%)		110/129 (85.3%)	902/1066 (84.6%)	181/205 (88.3%)	56/62 (90.3%)	0.37
Fertilization rate (%)		68.05 ± 28.61	67.22 ± 27.12	66.34 ± 29.47	60.98 ± 28.49	0.35
Embryo quality score		1.90 ± 0.66	2.03 ± 0.71	1.96 ± 0.73	1.92 ± 0.77	0.27
Day of ET						
Day 3		52/84 (61.9)	486/763 (63.7)	91/140 (65.0)	39/50 (78.0)	
Day 5		32/84 (38.1)	277/763 (36.3)	49/140 (35.0)	11/50 (22.0)	0.22
Embryos transferred (*n*)		1.69 ± 0.64	1.74 ± 0.62	1.73 ± 0.69	1.68 ± 0.59	0.81
Blastulation rate (%)		53.57 ± 23.09	56.57 ± 22.46	53.07 ± 20.75	52.65 ± 29.47	0.46

Notes: Data presented as mean ± SD, *n* (%) or proportion (%). ^a^ Pairwise comparison revealed a statistically significant difference between the second and the fourth BMI categories.

**Table 3:** Pregnancy outcomes by female BMI.

Variable	>18.50	18.50–24.99	25.0–29.99	≥30
Ongoing pregnancy	1.15 (0.67–1.96)	1.00	1.06 (0.68–1.66)	1.01 (0.49–2.06)
Miscarriage rate	1.37 (0.42–4.47)	1.00	2.24 (0.86–5.84)	4.75 (0.70–32.37)
Live birth	0.54 (0.17–1.79)	1.00	0.51 (0.18–1.41)	0.17 (0.02–1.31)
Gestational age (weeks)	37.89 ± 1.87	37.74 ± 3.03	37.59 ± 2.80	37.03 ± 4.41
Birth weight (g)	2887 ± 586	3053 ± 613	2932 ± 756	3131 ± 1186

Notes: Data presented as mean ± SD or adjusted OR (95% CI) (BMI category 18.50–24.99 was used as reference category). No statistical difference was observed for the reported outcomes across different BMI categories.

## References

[1] Ozcan-Dag Z., Oguzturk O., Isik Y., Turkel Y., Bulcun E. (2015). Personality profile in patients with polycystic ovary syndrome. Gynecol. Endocrinol..

[2] Haslan D.W., James W.P. (2005). Obesity. Lancet.

[3] Fakhouri T.H., Ogden C.L., Carroll M.D., Kit B.K., Flegal K.M. (2012). Prevalence of obesity among older adults in the United States, 2007–2010. NCHS Data Brief.

[4] Vahratian A. (2009). Prevalence of overweight and obesity among women of childbearing age. Mater. Child Health J..

[5] ISTAT Italia in Cifre. http://www.istat.it/it/files/2011/06/Italia_in_cifre_20132.pdf.

[6] Lashen H., Fear K., Sturdee D.W. (2004). Obesity is associated with increased risk of first trimester and recurrent miscarriage: Matched case-control study. Hum. Reprod..

[7] Weiss J.L., Malone F.D., Emig D., Ball R.H., Nyberg D.A., Comstock C.H., Saade G., Eddleman K., Carter S.M., Craigo S.D. (2004). Obesity, obstetric complications and cesarean delivery rate—A population-based screening study. Am. J. Obstet. Gynecol..

[8] Rajasingam D., Seed P.T., Briley A.L., Shennan A.H., Poston L. (2009). A prospective study of pregnancy outcome and biomarkers of oxidative stress in nulliparous obese women. Am. J. Obstet. Gynecol..

[9] Hartz A.J., Rupley D.C., Rimm A.A. (1984). The association of girth measurements with disease in 32,856 women. Am. J. Epidemiol..

[10] Douchi T., Kuwahata R., Yamamoto S., Oki T., Yamasaki H., Nagata Y. (2002). Relationship of upper body obesity to menstrual disorders. Acta Obstet. Gynecol. Scand..

[11] Talmor A., Dunphy B. (2015). Female obesity and infertility. Best Pract. Res. Clin. Obstet. Gynaecol..

[12] Gosman G.G., Katcher H.I., Legro R.S. (2006). Obesity and the role of gut and adipose hormones in female reproduction. Hum. Reprod. Update.

[13] Levens E.D., Skarulis M.C. (2008). Assessing the role of endometrial alteration amongobese patients undergoing assisted reproduction. Fertil. Steril..

[14] Dechaud H., Anahory T., Reyftmann L., Loup V., Hamamah S., Hedon B. (2006). Obesity does not adversely affect results in patients who are undergoing *in vitro* fertilization and embryo transfer. Eur. J. Obstet. Gynecol. Reprod. Biol..

[15] Pinborg A., Gaarslev C., Hougaard C.O., Nyboe Andersen A., Andersen P.K., Boivin J., Schmidt L. (2011). Influence of female bodyweight on IVF outcome: A longitudinal multicentre cohort study of 487 infertile couples. Reprod. Biomed. Online.

[16] Fedorcsák P., Dale P.O., Storeng R., Ertzeid G., Bjercke S., Oldereid N., Omland A.K., Abyholm T., Tanbo T. (2004). Impact of overweight and underweight on assisted reproduction treatment. Hum. Reprod..

[17] Ozekinci M., Seven A., Olgan S., Sakinci M., Keskin U., Akar M.E., Ceyhan S.T., Ergun A. (2015). Does obesity have detrimental effects on IVF treatment outcomes?. BMC Women’s Health.

[18] Caillon H., Fréour T., Bach-Ngohou K., Colombel A., Denis M.G., Barrière P., Masson D. (2015). Effects of female increased body mass index on *in vitro* fertilization cycles outcome. Obes. Res. Clin. Pract..

[19] Koning A.M., Mutsaerts M.A., Kuchenbecker W.K., Broekmans F.J., Land J.A., Mol B.W., Hoek A. (2012). Complications and outcome of assisted reproduction technologies in overweight and obese women. Hum. Reprod..

[20] Bellver J., Martínez-Conejero J.A., Labarta E., Alamá P., Melo M.A.B., Remohí J., Pellicer A., Horcajadas J.A. (2011). Endometrial gene expression in the window of implantation is altered in obese women especially in association with polycystic ovary syndrome. Fertil. Steril..

[21] Matalliotakis I., Cakmak H., Sakkas D., Mahutte N., Koumantakis G., Arici A. (2008). Impact of body mass index on IVF and ICSI outcome: A retrospective study. Reprod. Biomed. Online.

[22] Moragianni V.A., Jones S.M.L., Ryley D.A. (2012). The effect of body mass index on the outcomes of first assisted reproductive technology cycles. Fertil. Steril..

[23] Schliep K.C., Mumford S.L., Ahrens K.A., Hotaling J.M., Carrell D.T., Link M., Hinkle S.N., Kissell K., Porucznik C.A., Hammoud A.O. (2015). Effect of male and female body mass index on pregnancy and live birth success after *in vitro* fertilization. Fertil. Steril..

[24] Robker R.L., Akison L.K., Bennett B.D., Thrupp P.N., Chura L.R., Russell D.L., Lane M., Norman R.J. (2009). Obese women exhibit differences in ovarian metabolites, hormones, and gene expression compared with moderate-weight women. J. Clin. Endocrinol. Metab..

[25] World Health Organization BMI Classification. http://apps.who.int/bmi/index.jsp?introPage=intro_3.html.

[26] Rubino P., Viganò P., Luddi A., Piomboni P. (2015). The ICSI procedure from past to future: A systematic review of the more controversial aspects. Hum. Reprod. Update.

[27] Corti L., Papaleo E., Pagliardini L., Rabellotti E., Molgora M., La Marca A., Vigano P., Candiani M. (2013). Fresh blastocyst transfer as a clinical approach to overcome the detrimental effect of progesterone elevation at hCG triggering: A strategy in the context of the Italian law. Eur. J. Obstet. Gynecol. Reprod. Biol..

[28] Papaleo E., Corti L., Vanni V.S., Pagliardini L., Ottolina J., De Michele F., La Marca A., Viganò P., Candiani M. (2014). Basal progesterone level as the main determinant of progesterone elevation on the day of hCG triggering in controlled ovarian stimulation cycles. Arch. Gynecol. Obstet..

[29] Kinzer D.R., Barrett C.B., Powers R.D. (2008). Prognosis for clinical pregnancy and delivery after total fertilization failure during conventional *in vitro* fertilization or intracytoplasmic sperm injection. Fertil. Steril..

[30] Restelli L., Paffoni A., Corti L., Rabellotti E., Mangiarini A., Viganò P., Somigliana E., Papaleo E. (2014). The strategy of group embryo culture based on pronuclear pattern on blastocyst development: A two center analysis. J. Assist. Reprod. Genet..

[31] Paffoni A., Ferrari S., Viganò P., Pagliardini L., Papaleo E., Candiani M., Tirelli A., Fedele L., Somigliana E. (2014). Vitamin D deficiency and infertility: Insights from *in vitro* fertilization cycles. J. Clin. Endocrinol. Metab..

[32] Practice Committee of the American Society for Reproductive Medicine, Practice Committee of the Society for Assisted Reproductive Technology (2009). Guidelines on number of embryos transferred. Fertil. Steril..

[33] Alpha Scientists in Reproductive Medicine, ESHRE Special Interest Group of Embryology (2011). The Istanbul consensus workshop on embryo assessment: Proceedings of an expert meeting. Hum. Reprod..

[34] G*Power: Statistical Power Analyses for Windows and Mac. http://www.psycho.uni-duesseldorf.de/abteilungen/aap/gpower3/.

[35] Vanni V.S., Vigano P., Somigliana E., Papaleo E., Paffoni A., Pagliardini L., Candiani M. (2014). Vitamin D and assisted reproduction technologies: Current concepts. Reprod. Biol. Endocrinol..

[36] De Pergola G., Maldera S., Tartagni M., Pannacciulli N., Loverro G., Giorgino R. (2006). Inhibitory effect of obesity on gonadotropin, estradiol, and inhibin B levels in fertile women. Obesity.

[37] Brewer C.J., Balen A.H. (2010). The adverse effects of obesity on conception and implantation. Reproduction.

[38] Orvieto R., Nahum R., Meltcer S., Homburg R., Rabinson J., Anteby E.Y., Ashkenazi J. (2009). Ovarian stimulation in polycystic ovary syndrome patients: The role of body mass index. Reprod. Biomed. Online.

[39] Awartani K.A., Nahas S., Al Hassan S.H., Al Deery M.A., Coskun S. (2009). Infertilitytreatment outcome in sub groups of obese population. Reprod. Biol. Endocrinol..

[40] Dokras A., Baredziak L., Blaine J., Syrop C., VanVoorhis B.J., Sparks A. (2006). Obstetricoutcomes after *in vitro* fertilization in obese and morbidly obese women. Obstet. Gynecol..

[41] Bellver J., Melo M.A., Bosch E., Serra V., Remohí J., Pellicer A. (2007). Obesity and poor reproductive outcome: The potential role of the endometrium. Fertil. Steril..

[42] Robker R.L. (2008). Evidence that obesity alters the quality of oocytes and embryos. Pathophysiology.

[43] Carmina E., Lobo R.A. (1997). Gonadotrophin-releasing hormone agonist therapy for hirsutism is as effective as high dose cyproterone acetate but results in a longer remission. Hum. Reprod..

[44] Carmina E. (2003). Genetic and environmental aspect of polycystic ovary syndrome. J. Endocrinol. Investig..

[45] Jungheim E.S., Schoeller E.L., Marquard K.L., Louden E.D., Schaffer J.E., Moley K.H. (2010). Diet-induced obesity model: Abnormal oocytes and persistent growth abnormalities in the offspring. Endocrinology.

[46] Grindler N.M., Moley K.H. (2013). Maternal obesity, infertility and mitochondrial dysfunction: Potential mechanisms emerging from mouse model systems. Mol. Hum. Reprod..

[47] Shah D.K., Missmer S.A., Berry K.F., Racowsky C., Ginsburg E.S. (2011). Effect of obesity on oocyte and embryo quality in women undergoing *in vitro* fertilization. Obstet. Gynecol..

[48] Bellver J., Ayllon Y., Ferrando M., Melo M., Goyri E., Pellicer A., Remohì J., Meseguer M. (2010). Female obesity impairs *in vitro* fertilization outcome without affecting embryo quality. Fertil. Steril..

[49] Lintsen A.M., Pasker-de Jong P.C., de Boer E.J., Burger C.W., Jansen C.A., Braat D.D., van Leeuwen F.E. (2005). Effects of subfertility cause, smoking and body weight on the success rate of IVF. Hum. Reprod..

[50] Jungheim E.S., Lanzendorf S.E., Odem R.R., Moley K.H., Chang A.S., Ratts V.S. (2009). Morbid obesity is associated with lower clinical pregnancy rates after *in vitro* fertilization in women with polycystic ovary syndrome. Fertil. Steril..

[51] Vilarino F.L., Bianco B., Christofolini D.M., Barbosa C.P. (2010). Impact of body mass index on *in vitro* fertilization outcomes. Rev. Bras. Ginecol. Obstet..

[52] Metwally M., Li T.C., Ledger W.L. (2007). The impact of obesity on female reproductive function. Obes. Rev..

[53] Hamilton-Fairley D., Kiddy D., Watson H., Paterson C., Franks S. (1992). Association of moderate obesity with a poor pregnancy outcome in women with polycystic ovary syndrome treated with low dose gonadotrophin. Br. J. Obstet. Gynaecol..

[54] Wang J.X., Davies M.J., Norman R.J. (2002). Obesity increases the risk of spontaneous abortion during infertility treatment. Obes. Res..

[55] Maheshwari A., Stofberg L., Bhattacharya S. (2007). Effect of overweight and obesity on assisted reproductive technology—A systematic review. Hum. Reprod. Update.

[56] Pandey S., Pandey S., Maheshwari A., Bhattacharya S. (2010). The impact of female obesity on the outcome of fertility treatment. J. Hum. Reprod. Sci..

[57] Jungheim E.S., Schon S.B., Schulte M.B., DeUgarte D.A., Fowler S.A., Tuuli M.G. (2013). IVF outcomes in obese donor oocyte recipients: A systematic review and metaanalysis. Hum. Reprod..

[58] Luke B., Brown M.B., Stern J.E., Missmer S.A., Fujimoto V.Y., Leach R. (2011). Racial and ethnic disparities in assisted reproductive technology pregnancy and live birth rates within body mass index categories. Fertil. Steril..

[59] Roque M. (2015). Freeze-all policy: Is it time for that?. J. Assist. Reprod. Genet..

[60] Aflatoonian A., Oskouian H., Ahmadi S., Oskouian L. (2010). Can fresh embryo transfers be replaced by cryopreserved-thawed embryo transfers in assisted reproductive cycles? A randomized controlled trial. J. Assist. Reprod. Genet..

[61] Shapiro B.S., Daneshmand S.T., Garner F.C., Aguirre M., Hudson C., Thomas S. (2011). Evidence of impaired endometrial receptivity after ovarian stimulation for *in vitro* fertilization: A prospective randomized trial comparing fresh and frozen-thawed embryo transfer in normal responders. Fertil. Steril..

[62] Maheshwari A., Pandey S., Shetty A., Hamilton M., Bhattacharya S. (2012). Obstetric and perinatal outcomes in singleton pregnancies resulting from the transfer of frozen thawed *versus* fresh embryos generated through *in vitro* fertilization treatment: A systematic review and metaanalysis. Fertil. Steril..

[63] Pelkonen S., Hartikainen A.L., Ritvanen A., Koivunen R., Martikainen H., Gissler M., Tiitinen A. (2014). Major congenital anomalies in children born after frozen thawed embryo transfer: A cohort study 1995–2006. Hum. Reprod..

[64] Pinborg A., Henningsen A.A., Loft A., Malchau S.S., Forman J., Andersen A.N. (2014). Large baby syndrome in singletons born after frozen embryo transfer (FET): Is it due to maternal factors or the cryotechnique?. Hum. Reprod..

